# Queen of Spices, Cardamom (*Elettaria cardamomum* (L.) Maton, Zingiberaceae)—In Vitro Assessment of Biological Potential

**DOI:** 10.3390/molecules31142430

**Published:** 2026-07-11

**Authors:** Maja Hitl, Katarina Radovanović, Katarina Urumović, Blagoje Prpa, Nebojša Kladar

**Affiliations:** 1 Department of Pharmacy, Faculty of Medicine Novi Sad, University of Novi Sad, Hajduk Veljkova 3, 21000 Novi Sad, Serbia; katarina.radovanovic@mf.uns.ac.rs (K.R.); katarina.bijelic@mf.uns.ac.rs (K.U.); blagoje.prpa@mf.uns.ac.rs (B.P.); nebojsa.kladar@mf.uns.ac.rs (N.K.); 2 Center for Medical and Pharmaceutical Investigations and Quality Control (CEMPhIC), Faculty of Medicine Novi Sad, University of Novi Sad, 21000 Novi Sad, Serbia

**Keywords:** *Elettaria cardamomum*, true cardamom, essential oil, GC-MS, antioxidant, anti-inflammatory, antihyperglycemic, antimicrobial

## Abstract

Cardamom represents one of the world’s most famous spices, which is also applied in the traditional medicine of numerous cultures. Modern research aims to elucidate its therapeutic potential in various diseases. The aim of this study was to investigate the chemical composition of essential oil of cardamom seeds, as well as to assess the biological and pharmacological potential of aqueous and ethanolic extracts. The essential oil composition was evaluated using gas chromatography coupled to mass spectrometry. Aqueous and ethanolic extracts were analyzed for the total phenolic and flavonoid content, and further evaluated in vitro regarding antioxidant, anti-inflammatory and antihyperglycemic activity, and additionally antimicrobial activity for the ethanolic extract. The essential oil was abundant in monoterpenes, with *α*-terpinyl acetate as a predominant compound. The content of total phenolics and flavonoids in extracts was low. Antioxidant potential was moderate. Extracts displayed potent activity in tests of anti-inflammatory potential, with the ethanolic extract being more active. Regarding the antihyperglycemic activity, the extracts displayed inhibitory activity against *α*-amylase and no activity against *α*-glucosidase. Antimicrobial activity was observed against *Candida albicans* and *Prototheca zopfii* in high concentrations. The present study suggests preliminary in vitro potential of cardamom in mediating carbohydrate metabolism and pro-inflammatory conditions.

## 1. Introduction

Cardamom, *Elettaria cardamomum* (L.) Maton, Zingiberaceae, also known as the “queen of spices”, represents the world’s third most expensive spice (after saffron and vanilla). This spice has a long history of traditional use, and although it is considered native to the Indian subcontinent, it is mentioned in many early civilizations, such as Ancient India, Egypt, Greece, and Rome. In culinary application, it is frequently added to coffee and tea, pastries, cakes, and spice blends for meat and vegetarian dishes [1,2]. Due to its high price, the authentic species (commonly referred to as true, small, or green cardamom) is often substituted and adulterated with species from the genera *Amomum* and *Aframomum*. The mentioned species have a similar aroma to true cardamom; however, in addition to botanical differences, a major distinction is seen in the phytochemical and phytotherapeutic profile [3].

The pleasant taste and aroma of cardamom are attributed to the presence of essential oil (EO). The essential oil is located in the seeds, which are protected by capsule pods. The content of EO in cardamom seeds can be classified as very high, reaching an astonishing 15% in some cultivars. Besides EO, other groups of secondary metabolites have been reported in cardamom, including alkaloids, flavonoids, anthocyanins and tannins [4,5]. Although EO can be considered the most abundant and most valuable type of plant extract, cardamom can be used in its entirety, in powdered form as a spice added to food and beverages, or for the preparation of macerates and decoctions; thus, other compounds besides those present in EO can be considered important for the plant’s biological activity [5].

Traditional medicine describes numerous applications and benefits of cardamom’s use. It is believed to aid in various digestive disorders, respiratory diseases, and kidney and heart problems. The positive effect on the digestive tract is especially prominent; cardamom is used for various disorders, including both diarrhea and constipation, and the application of the herb in dental and periodontal disorders is frequently reported. Cardamom is also seen as a remedy for epilepsy, headaches, and as an antidote in snakebites and scorpion stings [4].

Modern research focuses on investigating the traditionally reported uses of medicinal herbs and aims to elucidate the mechanism of action of used herbal preparations. Clinical studies conducted in humans have also suggested the potential of cardamom in treating oxidative stress and inflammation-related disorders, as it reduces prooxidative and proinflammatory mediators [6]. A systematic review from 2022 suggested that cardamom may improve the metabolism of sugars, as it was seen to regulate homeostatic model assessment for insulin resistance (HOMA-IR) and glycated hemoglobin (HbA1C), two parameters often investigated in chronic diseases associated with impaired carbohydrate metabolism [7]. Finally, numerous in vitro studies on antimicrobial activity have confirmed its potential against various microorganisms, including foodborne pathogens [8,9] and those associated with periodontal diseases [10].

Although some previously published papers have tested cardamom’s potential in vitro, relatively few studies have comprehensively evaluated its biological activities and explored the mechanisms responsible for these effects. This research aimed to investigate the chemical composition of cardamom’s essential oil and extracts, as well as to evaluate the in vitro biological potential of aqueous and ethanolic extracts of cardamom seeds.

## 2. Results and Discussion

### 2.1. Chemical Analysis of Essential Oil

EO represents a complex mixture of aromatic, lipophilic compounds widely used in the pharmaceutical, food, and cosmetic industries. Considering that cardamom seeds primarily represent an aromatic herbal drug, the EO content, as well as the compounds present in it, are often investigated and applied in various industries.

The extraction yield was 2.06% (*m*/*m*). Other studies report much higher yields, with 22 investigated accessions of cardamom from India containing 4.5–9.5% of EO, isolated by hydrodistillation [11], while a study reporting combined methods of extraction, with various investigated parameters, showed that the yield varied between 2.52 and 4.43% [12].

The most abundant group of compounds in EO consisted of monoterpenes, with oxygenated monoterpenes representing a total of 92.40% of identified compounds in EO. Sesquiterpenes were less abundant. The details are presented in Table 1.

Previous research investigated the content of cardamom’s EO. A study by Noumi et al., 2018 [3] found that the predominant group of compounds was oxygenated monoterpenes (63%), followed by a significantly higher proportion of monoterpene hydrocarbons (36.9%) than in the present study. Additionally, in contrast to the present study, no sesquiterpenes were detected in those samples. A study by Ivanović et al., 2021 [13] found that 93.04% of detected compounds were monoterpenes, while only 0.11% of compounds were sesquiterpenes. A higher percentage of sesquiterpenes was found in another study, with >9% of total detected compounds; still, monoterpenes were the most abundant group in EO [14].

Regarding the specific single compounds (Table 2 and Supplementary Figure S1), the most abundant compound in EO was α-terpinyl acetate (57.50% of total content), followed by oxygenated monoterpenes eucalyptol (14.63%) and α-terpineol (6.50%). Several other studies also found α-terpinyl acetate as the main component (29.9–55.99%), followed by eucalyptol as the second most abundant [11,13,14,15]. However, some studies report opposite findings, with eucalyptol being the most abundant compound (around 55%), followed by α-terpinyl acetate [3,16]. It is interesting to point out that different extraction techniques can affect the composition of EO, and by varying experimental conditions, higher or lower proportions of certain compounds can be isolated [15,17].

### 2.2. Preliminary Chemical Characterization of Aqueous and Ethanolic Extracts

In addition to EO, it is possible to use various extracts, with herbal material used both before and after EO extraction. This type of use reflects the common mode of application of medicinal herbs and spices in households, e.g., teas, beverages, or culinary use with wine or other types of alcoholic beverages.

The extraction yield was similar for both types of extracts: 8.34 ± 0.42% for aqueous extract and 8.93 ± 0.65% for ethanolic extract (no statistically significant differences were recorded, Z = −0.87, *p* = 0.4).

The content of total phenolics was 5.21 ± 0.26 mg GAE/g DE (milligrams of gallic acid equivalents (GAE) per gram of dry extract (DE)) for aqueous extract and 9.27 ± 0.21 mg GAE/g DE for ethanolic extract. In both cases, the total phenolic content can be classified as low. This is in accordance with previous studies [13,18,19], where it was found that the use of different solvents (water, 50% ethanol, 80% ethanol, 80% methanol and pure methanol) does not result in differences in extracts’ total phenolic content, with all obtained extracts containing low amounts of phenolics (˂5 mg GAE/g DE). However, this is in contrast with other previously published results, which report approximately three times higher content of phenolics than in the present study—33.45 and 31.06 mg GAE/g DE for ethanolic and methanolic extracts, respectively [20].

Regarding the content of total flavonoids, it can be concluded that these compounds were present in trace amounts, with 0.47 ± 0.15 mg QE/g DE (milligrams of quercetin equivalents (QE) per gram of DE) in aqueous extracts and 0.57 ± 0.09 mg QE/g DE in ethanolic extract. These results are in accordance with a study by Przygodzka et al., 2014 [21], where both total phenolic and total flavonoid content were characterized as very low, with the additional observation that flavonoid content was higher when ethanol was used as a solvent (rather than a 50:50 mixture of ethanol and water). Again, previous results [20] suggest much higher amounts of total flavonoids, with 67.38 and 58.41 mg of rutin equivalents per g of extract. However, it is important to point out that this research detected higher amounts of flavonoids than phenolics in the investigated extracts, thus indicating the noticed differences may be the result of different methods for determining the content of these compounds, rather than illustrating the differences between various samples of herbal material.

### 2.3. Antioxidant Activity

Herbal extracts obtained using different solvents are often recognized for their antioxidant potential. This supports the use of medicinal plants in the treatment and prevention of various conditions and diseases, primarily those in which pro-oxidant mediators are involved in the pathogenesis or affect clinical outcomes. In the present study, ethanolic and aqueous extracts were evaluated since they are easily obtained with low-toxicity solvents [22].

The results of antioxidant activity testing are presented in Table 3. Cardamom extracts display antioxidant activity in the test of neutralization of 2,2-diphenyl-1-picrylhydrazyl (DPPH) radicals and the test of ferric reducing antioxidant power (FRAP); however, their activity can be characterized as moderate. In both cases, the ethanolic extract was more potent than the aqueous one.

An aqueous extract was evaluated in a study by Alkhalifah et al., 2022 [23], and it displayed somewhat lower activity in the DPPH assay (with RSC_50_ (radical scavenging concentration) of 290.8 μg/mL, as opposed to 162.96 μg/mL in the present study). A previous study by Tarfaoui et al., 2022 [20] also tested the antioxidant activity of various extracts of cardamom using the same tests as in the present study. Regarding the DPPH test, the concentration needed to neutralize the DPPH radicals was RSC_50_ = 423 μg/mL for ethanolic and RSC_50_ = 731 μg/mL for methanolic extract (aqueous extract was not tested in the mentioned research), these values being much higher than those recorded in the present research. Although the study by Ali et al., 2020 [18] did not report RSC_50_, the results suggest lower antioxidant potential in the DPPH test, especially when compared with extracts of other plants in the Zingiberaceae family.

As opposed to this, the present study reports lower antioxidant potential in the FRAP test (10.29 and 13.63 mg AAE/g DE (milligrams of ascorbic acid equivalents (AAE) per gram of DE) for aqueous and ethanolic extracts, respectively, while a previous study [20] reports higher potential of ethanolic and methanolic extracts (approximately 90 mg AAE/g DE). Another study found even lower antioxidant potential (˂5 mg Fe^2+^/g DE) of cardamom aqueous, ethanolic and methanolic extracts than that reported in the present study [13].

### 2.4. Anti-Inflammatory Activity

In addition to antioxidant potential, herbal extracts can be evaluated for anti-inflammatory potential. Pro-inflammatory mediators are involved in the pathogenesis of numerous diseases and conditions; thus, natural products that modulate inflammatory response represent valuable sources of bioactive compounds interesting for researchers investigating human health and aforementioned entities [24,25].

**Figure 1:**
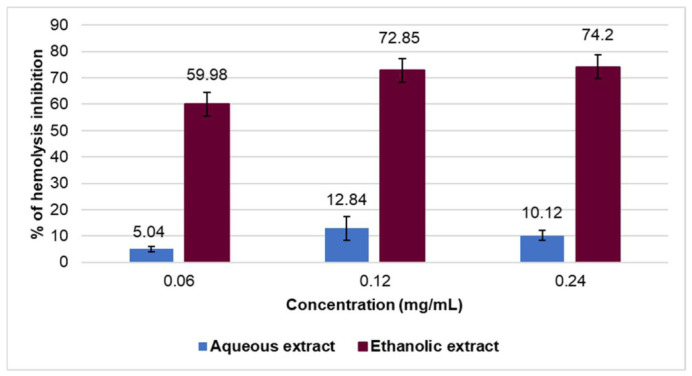
The hemolysis inhibition activity of the studied cardamom extracts.

The anti-inflammatory potential of cardamom was investigated using an in vitro thermohemolysis inhibition assay, with different concentrations of cardamom extract (Figure 1). The aqueous extract showed only minor inhibition of the induced process. In contrast, ethanolic extract displayed anti-inflammatory activity at 0.06 mg/mL and 0.12 mg/mL; however, at increasing concentrations, it displayed only a minor increase in activity, suggesting a plateau in activity. Diclofenac sodium, a conventional drug which displays anti-inflammatory properties, applied as a positive control, also displayed anti-inflammatory activity, with 0.1 mg/mL inhibiting 87.23 ± 4.43% of hemolysis.

Similar results were obtained in a study by Shahid et al., 2022 [19], where a single concentration of cardamom’s aqueous and methanolic extracts was evaluated in the same in vitro test of inhibition of thermohemolysis. The research results were similar to those obtained in the present study, where the methanolic extract inhibited 89.09%, and the aqueous extract inhibited 63.17% of the hemolysis process, with the positive control, diclofenac sodium, displaying 73.17% of inhibition. The mentioned study also confirmed that extracts obtained using alcohols show greater potential in this anti-inflammatory test; however, the differences were not as prominent as in the present study. It is important to point out that the aforementioned assay represents only one in vitro method, which can be used for preliminary assessment of anti-inflammatory properties, and that it may not reflect cellular mechanisms of bioactive compounds in mediating inflammatory response. Many other studies showed the anti-inflammatory potential of cardamom, including clinical studies in which inflammatory biomarkers were evaluated [26,27].

### 2.5. Antihyperglycemic Activity

Antihyperglycemic activity was evaluated in the test of inhibition of α-amylase and α-glucosidase activity. These enzymes are responsible for the initial stage of carbohydrate intestinal metabolism and the postprandial spike in blood sugar levels; thus, in vitro modulation of these enzymes may contribute to a better understanding of strategies aimed at managing diabetes mellitus type 2 and related clinical conditions preceding the disease, e.g., insulin resistance [28].

**Figure 2:**
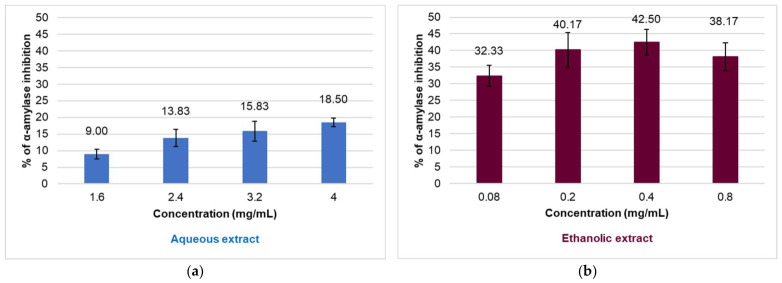
The inhibition of α-amylase activity by (**a**) aqueous and (**b**) ethanolic cardamom extracts.

Regarding the anti-α-amylase activity, the results of the performed test are given in Figure 2a,b. As can be noticed, aqueous extract showed only minor inhibition of the enzyme (not reaching the inhibition of 50% of the enzyme’s activity), while ethanolic extract showed inhibition of approximately 40% of the enzyme’s activity at applied concentrations (which were even lower than the ones used in the test with aqueous extracts). In contrast, the positive control, acarbose, displayed potent anti-α-amylase activity in much lower concentrations, with 4.23 ± 0.33 μg/mL inhibiting 50% of the enzyme’s activity, indicating higher potency than herbal extracts.

A study by Ahmed et al., 2017 [29] evaluated aqueous and methanolic extracts of cardamom in an α-amylase inhibitory assay. The study found better activity of the aqueous extract (it inhibited approximately 80% of the enzyme’s activity, contrasting the results of the present study), while the methanolic extract displayed a similar level of activity to the ethanolic extract in the present study, with inhibition of approximately 40% of α-amylase activity. Research by Al-Yousef et al., 2021 [30] tested several concentrations of cardamom aqueous extract, determining IC_50_ (the concentration inhibiting 50% of the enzyme’s activity) to be 220.5 µg/mL, this value being similar to the ethanolic extract of the present study (200 µg/mL inhibiting 40.17% of α-amylase’s activity) rather than the aqueous one. These studies, as well as the present one, suggest that cardamom extract can exhibit antihyperglycemic activity by modifying the activity of α-amylase. It is important to point out that various factors, including extraction solvent, origin of herbal drug, assay protocol, concentration range of tested extracts, etc., can contribute to differences in in vitro activity, which can be noticed among reported research.

**Figure 3:**
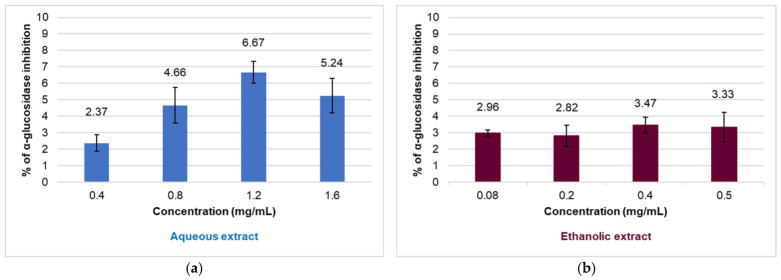
The inhibition of α-glucosidase activity by (**a**) aqueous and (**b**) ethanolic cardamom extracts.

Cardamom extracts were also tested for their ability to inhibit the activity of *α*-glucosidase. Figure 3a,b represent the percent of inhibition at corresponding concentrations for aqueous and ethanolic extracts, respectively.

Based on the low levels of inhibition of α-glucosidase, it can be assumed that cardamom extracts do not affect the activity of this enzyme. The positive control, acarbose, displayed activity against α-glucosidase, with 44.67 ± 1.22 μg/mL inhibiting 50% of the enzyme’s activity.

Results of the present study are in accordance with the results obtained in a study by Ahmed et al. 2017 [29], where aqueous and methanolic extracts of cardamom were investigated, and both types of extracts inhibited less than 15% of the enzyme’s activity.

### 2.6. Antimicrobial Activity

Antimicrobial resistance represents a growing therapeutic challenge. Besides the commonly recorded resistance of bacteria to antibiotics, other types of microbes also show emerging resistance to conventionally used drugs. Additionally, the majority of microbes can be causative agents of both human and animal infections, as is the case with the fungus *Candida albicans* and microalga *Prototheca zopfii* [31].

Relatively high concentrations of cardamom extracts were required for achieving antimicrobial effects in both *Candida* and *Prototheca*. Comparison with nystatin, used as a positive control, suggests limited potency of cardamom, and the tested extracts displayed considerably lower antimicrobial potency in this in vitro screening. The results are presented in Table 4.

These results are somewhat in accordance with the results by Tarfaoui et al., 2022 [20], where anticandidal activity was tested in another type of assay, the disk diffusion test, in which no activity against *C. albicans* or *Candida tropicalis* was recorded when ethanolic and methanolic extracts of cardamom were used. Another study compared ethanolic and acetonic extracts of *E. cardamomum*, also in the disk diffusion test, showing that the acetonic extract was more potent than the ethanolic extract against *Candida* spp., further showing the same result in the biofilm-forming inhibition test [32]. To the best of our knowledge, no previous study has investigated the activity of cardamom against *Prototheca* spp. Future studies could evaluate various extracts (obtained by different solvents or their mixtures) and search for the mechanism of their activity.

### 2.7. Study Limitations and Additional Considerations

The presented study has several limitations. The chemical characterization of aqueous and ethanolic extracts is limited to a preliminary evaluation of TPC and TFC. Additional evaluation of single chemical compounds using an adequate chromatographic method was not performed. The main reason for such an approach was the low content of these bioactive compound groups, as determined by spectrophotometric methods. This suggests that the compounds responsible for recorded activities in assays belong to other group(s) of chemical compounds. However, without further analysis, active constituents remain unidentified.

Additionally, the present study used a single sample of cardamom, obtained from a local spices store. Differences between results of studies reported previously in the available literature, as well as between the present study and previously published papers, can be noticed. Several factors can contribute to these differences. Primarily, herbal drugs can differ due to various biotic and abiotic factors (e.g., soil composition, insolation, temperature, pollinators) affecting the plant’s synthesis of bioactive compounds. Further handling and storage of herbal drugs can also contribute to differences noticed in various studies, as harvest date, storage conditions and post-harvest handling may influence both essential oil composition and extract activity. Furthermore, methods used to obtain extracts (including EO) can affect the chemical constituents isolated from herbal material. This is most prominently seen in the use of various extraction solvents. Finally, various methods are employed in the assessment of biological activity. Different assay protocols can contribute to differences between investigated parameters; thus, reporting final concentrations tied to biological effects is required, as well as presenting a comparison of the investigated agent and a suitable positive control under the same experimental conditions. Precisely because of all the various factors that can affect the final chemical profile and biological activities (observed in vitro but also in clinical settings), it is important to perform various studies that investigate medicinal plants, including cardamom.

The present study has an important advantage. Aqueous *infusum* and ethanolic macerate are extracts obtained “mimicking” the preparations in household settings, as commonly used by patients and consumers of herbal drugs (including cardamom). By treating the freshly ground, powdered drug, the bioactive compounds are isolated in used extraction solvents. And while components of EO are predominantly volatile, at the same time, they display certain solubility in used extraction solvents (particularly ethanol), and the obtained extracts can contain both these volatile and other, non-volatile compounds. It is possible that their combination provides the biological effects recorded in the presented in vitro assays.

## 3. Materials and Methods

### 3.1. Herbal Drug

**Figure 4:**
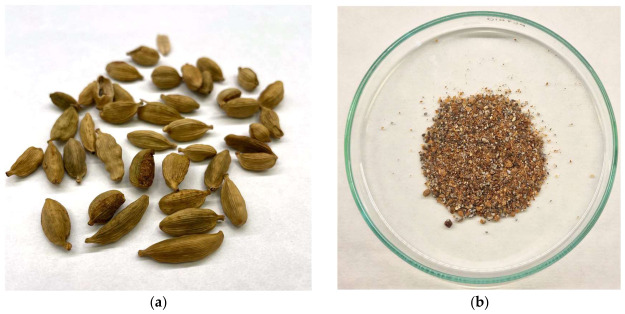
The appearance of herbal drug: (**a**) cardamom pods, before peeling and grinding; (**b**) powdered form, after peeling and grinding seeds.

In this investigation, the plant species *Elettaria cardamomum* (L.) Maton, Zingiberaceae was used. The herbal drug, in the form of whole pods, was purchased at a commercial store specializing in herbal teas, spices and other types of health foods. The herbal material was evaluated, and the identity of the species was confirmed by the Herbarium of the Department of Biology, Faculty of Sciences, University of Novi Sad, and a voucher specimen was deposited under No. 2-2134. The seeds of the plant were used as an herbal drug after peeling the protective layers of the pods. Subsequently, the drug was ground in an electric mill (FG electronics, Foshan, China), using pulse grinding, in order to avoid overheating of the herbal material (Figure 4).

### 3.2. Chemical Analysis of Essential Oil

EO was isolated from seeds by hydrodistillation, as described in the European Pharmacopoeia 10 [33]. The extraction yield was calculated based on the starting weight of dry ground cardamom seeds (%, *m*/*m*). Chemical composition was subsequently analyzed by gas chromatography with mass spectrometry (GC–MS), applying a standard semi-quantitative method based on relative peak area percentages. A sample of 20 µL of the obtained EO was diluted in 980 µL of hexane and further analyzed by a GC system (7890B GC System coupled to a 5977A MSD; Agilent Technologies, Waldbronn, Germany) with MS detection using an HP-5MS (30 m × 0.250 mm × 0.25 μm; Agilent Technologies) column. The initial oven temperature was 60 °C. The temperature was increased to 246 °C at a rate of 3 °C/min, maintained for 3 min, and then raised to 280 °C and maintained for 5 min. The temperature of the inlet was 220 °C, and split mode was used, 20:1. The MS was operated in scan mode (*m*/*z* = 30–550). The flow of helium was constant (1 mL/min), and the transfer line temperature was 285 °C. The identification of compounds present in EO was based on the comparison of relative retention indices (RI) and mass spectra with the NIST v14 spectral library [34], considering only compounds with a spectral match of at least 95%. This analysis was performed as a single-sample distillation and in a single run in the GC-MS system.

### 3.3. Preparation of Aqueous and Ethanolic Extracts and Preliminary Chemical Characterization

With the aim of using a traditional type of phytopreparation, which can be prepared by consumers in household settings, two types of extracts were prepared. The aqueous extract was obtained in the form of an *infusum* by treating the herbal drug with mildly heated water (~50 °C), and the ethanolic extract was prepared by maceration technique with 70% (*v*/*v*) ethanol. With the purpose of better utilization of the drug, a multiple extraction was performed; each extraction was performed using a 1:10 drug-to-solvent ratio, the extraction lasted 24 h in the dark, and the process was repeated a total of three times. Combined liquid extracts were filtered and evaporated to dryness. The extraction yield was calculated based on the starting weight of dry ground seeds of cardamom (%, *m*/*m*). Both extracts were reconstituted using water and further evaluated for in vitro antioxidant, anti-inflammatory, antihyperglycemic and antimicrobial potential. All experiments were performed in triplicate. All spectrophotometric measurements were performed on Shimadzu UV 1800 spectrophotometer (Shimadzu Corporation, Kyoto, Japan).

A preliminary chemical characterization of the extracts was performed by evaluating the content of total phenolics and total flavonoids, using previously described spectrophotometric methods [35]. Briefly, the content of phenolics was determined using Folin–Ciocalteu reagent (Merck, Darmstadt, Germany), and the absorbance of the developed complex was obtained at 760 nm. The content of total flavonoids was determined using Al^3+^-reagent (Sigma Aldrich, St. Louis, MO, USA), which, in reaction with flavonoids, forms a complex displaying characteristic absorbance properties at 430 nm. The amounts of total phenolics and flavonoids were calculated using standard calibration curves previously obtained for gallic acid and quercetin (Sigma Aldrich, St. Louis, MO, USA), respectively, under the same experimental conditions. The results are expressed in milligrams of gallic acid equivalents (GAE) per gram of dry extract (DE) (mg GAE/g DE) for total phenolics, and milligrams of quercetin equivalents (QE) per gram of DE (mg QE/g DE) for total flavonoids.

### 3.4. Antioxidant Activity

The testing of antioxidant activity was performed using the test of neutralization of DPPH radicals and the FRAP assay. Briefly, the DPPH test was performed using a previously described spectrophotometric method [36], where the disappearance of the purple color, as a result of DPPH radicals (Alfa Aesar, Haverhill, MA, USA) neutralization, was measured at 515 nm. Propyl gallate (Sigma Aldrich, St. Louis, MO, USA) was used as a positive control. The results were expressed as the concentration that scavenges 50% of free radicals (radical scavenging concentration, RSC_50_, in μg/mL). Regarding the FRAP test, the previously described spectrophotometric method [37] was used to estimate the ability of cardamom extract to reduce ferric to ferrous ions, at three concentration levels, and the intensity of ink-blue color was monitored at 593 nm. The results are expressed as milligrams of ascorbic acid (Sigma Aldrich, St. Louis, MO, USA) equivalents (AAE) per gram of DE (mg AAE/g DE).

### 3.5. Anti-Inflammatory Activity

Investigation of anti-inflammatory activity was performed in an experiment of inhibition of thermal-induced hemolysis. The assay is based on the hypothesis that added herbal extract may act as a membrane-stabilizing agent and subsequently prevent formation of inflammatory mediators. The test was performed according to the previously described procedure [38] with minor modifications. In brief, a 10% (*v*/*v*) suspension of human erythrocytes was used. A blood sample (citrate was used an anticoagulant) was taken from a healthy volunteer; ethical approval was given by the Ethical Committee of Faculty of MedicineNovi Sad, University of Novi Sad. The erythrocytes were washed three times with saline, and the suspension was reconstituted with isotonic phosphate buffer (pH 7.4). Different volumes of aqueous or ethanolic extract were mixed with 100 μL of erythrocyte suspension, and phosphate buffer was added up to a final volume of 3 mL. The mixture was incubated in a shaking water bath (54 °C, 20 min), and the thermohemolysis was stopped by immersion in cold water. The test tubes were centrifuged, and the absorbance of the supernatant was measured at 540 nm. Control tubes contained no herbal extract, and phosphate buffer was used as a blank. Diclofenac sodium (Sigma Aldrich, St. Louis, MO, USA) was used as a positive control. The results are expressed as % of hemolysis inhibition.

### 3.6. Antihyperglycemic Activity

Antihyperglycemic potential of cardamom extracts was evaluated in tests of inhibition of enzymes involved in carbohydrate metabolism, α-amylase and α-glucosidase, according to previously described procedures [36]. Regarding the α-amylase assay, the herbal extracts were mixed with commercially available starch complexed with “indicator color” (remazol brilliant blue) (Sigma Aldrich, St. Louis, MO, USA), porcine α-amylase (Sigma Aldrich, St. Louis, MO, USA) and phosphate buffer. After incubation for 10 min, the reaction was stopped by addition of acetic acid, the test tubes were centrifuged, and the absorbance of supernatants containing the indicator was measured spectrophotometrically at 595 nm. Control tubes contained no added herbal extract, and phosphate buffer was used as a blank. The results are expressed as % of α-amylase inhibition. In the test of α-glucosidase inhibition, the cardamom extracts were mixed with α-glucosidase (obtained from *Saccharomyces cerevisiae*) (Sigma Aldrich, St. Louis, MO, USA), reduced glutathione, substrate p-nitrophenyl-D-glucoside (Sigma Aldrich, St. Louis, MO, USA) and phosphate buffer. After incubation for 20 min, the reaction was stopped with concentrated sodium carbonate solution, and the absorbance of the yellow-colored solutions was measured spectrophotometrically at 400 nm. Control tubes contained no herbal extract, and phosphate buffer was used as a blank. The results are expressed as % of α-glucosidase inhibition. In both assays, acarbose (Sigma Aldrich, St. Louis, MO, USA) was used as a positive control.

### 3.7. Antimicrobial Activity

Testing was performed by using the microdilution method, as previously described [39]. Antimicrobial activity was tested against strains of fungus *Candida albicans* and microalgae *Prototheca zopfii*. The first strain of *C. albicans* was a clinical isolate (isolated from a human patient), while the second was American Type Culture Collection (ATCC) 24433 strain. Both strains of *P. zopfii* were clinical isolates, one originating from a human and the other from an animal patient. Microorganisms were cultivated in aerobic conditions, on Sabouraud dextrose agar plates (Torlak, Belgrade, Serbia) at 37 °C for 48 h. Microbial suspension was prepared using saline (McFarland standard 0.5), and its density was measured spectrophotometrically at 625 nm. The testing was performed using the double dilution method, with wells containing a microbial suspension, Sabouraud dextrose broth (Torlak, Belgrade, Serbia) and diluted cardamom extracts. Microtitration plates were incubated in aerobic conditions at 37 °C for 24 h. After this period, the contents of wells displaying no visible growth were transferred to Sabouraud dextrose agar plates and cultivated for another 24 h. The method also included control of sterility (wells contained no microbial suspension), control of validity (wells contained no cardamom extract) and positive control (wells contained nystatin (Hemofarm, Vršac, Serbia) instead of cardamom extract). The results were expressed as minimal concentration, which inhibited the growth of microorganisms (minimal inhibitory concentration, MIC, in mg/mL) and minimal fungicidal concentration (MFC), for *Candida* sp. (in mg/mL), or minimal algaecidal concentration (MAC), for *Prototheca* sp. (in mg/mL). Only the ethanolic extract was tested in this experiment.

### 3.8. Data Analysis

All data were analyzed using the software package Microsoft Excel for Windows, v. 2016, and Tibco Statistica, v. 13.5. The statistical significance of differences in the obtained results between the types of evaluated extracts was tested by Mann–Whitney U test and Kruskal–Wallis ANOVA, whereas the level of significance was set at *p* = 0.05.

## 4. Conclusions

Cardamom seeds represent an herbal drug containing essential oil. The EO is abundant in valuable monoterpene compounds with previously demonstrated biological and pharmacological potential. Furthermore, cardamom ethanolic and aqueous extracts exhibited notable activity in some of the applied in vitro tests; namely, the ethanolic extract displayed anti-inflammatory activity and α-amylase inhibitory activity, suggesting the need for further studies regarding antihyperglycemic potential. This research provides preliminary data and supports further in vitro and in vivo investigation of cardamom in inflammatory diseases and conditions involving elevated glycemia.

## Figures and Tables

**Table 1:** The content of the compound groups in cardamom’s essential oil.

Group of Compounds	Content (%)
monoterpene hydrocarbons	3.87
aromatic monoterpene hydrocarbons	0.22
oxygenated monoterpenes	92.40
aromatic oxygenated monoterpenes	0.10
sesquiterpene hydrocarbons	0.42
oxygenated sesquiterpenes	1.76

**Table 2:** The content of compounds (%) in cardamom’s essential oil.

RT	RI	Compound	%
7.35	929	*α*-pinene	0.19
8.53	974	sabinene	0.65
8.67	979	*β*-pinene	0.10
9.04	991	*β*-myrcene	0.45
9.10	993	2,3-dehydro-1,8-cineole	0.28
9.98	1017	*α*-terpinene	0.24
10.27	1023	*m*-cymene	0.22
10.44	1030	D-limonene	1.19
10.60	1032	eucalyptol	14.63
11.53	1060	*γ*-terpinene	0.56
12.10	1086	furan linalool oxide	0.52
12.70	1088	terpinolene	0.50
13.17	1099	linalool	5.01
14.04	1140	*p*-2-menthen-1-ol	0.17
15.93	1167	*α*-phellandren-8-ol	0.37
16.39	1182	terpinen-4-ol	2.41
16.71	1183	*p*-cymen-8-ol	0.10
17.03	1189	*α*-terpineol	6.50
18.15	1217	carveol	0.34
19.03	1240	neral	0.10
19.18	1246	D-carvone	0.10
19.65	1257	linalyl acetate	3.20
20.27	1276	citral	0.12
22.26	1315	*δ*-terpinyl acetate	0.18
23.84	1350	*α*-terpinyl acetate	57.50
24.19	1364	neryl acetate	0.20
24.98	1382	geranyl acetate	0.63
27.11	1427	*α*-terpinyl propionate	0.11
29.17	1489	*β*-selinene	0.26
30.23	1513	*γ*-cadinene	0.16
32.13	1564	E-nerolidol	1.76
		total content of identified components	98.77

RT—retention time, RI—retention index.

**Table 3:** The antioxidant activity of cardamom extracts.

Test	Aqueous Extract	Ethanolic Extract	Propyl Gallate
DPPH (RSC_50_, in μg/mL)	162.96 ± 12.32 ^a^	91.49 ± 5.32 ^a,c^	0.63 ± 0.04 ^b,c^
FRAP (mg AAE/g DE)	10.29 ± 0.87 ^a^	13.63 ± 2.36 ^a^	NA

The different lowercase letters denote statistically significant differences (*p* < 0.05) between tested samples; DPPH—2,2-diphenyl-1-picrylhydrazyl, RSC_50_—concentration which can scavenge 50% of free radicals, FRAP—ferric reducing antioxidant power, AAE—ascorbic acid equivalents, DE—dry extract, NA—not applicable.

**Table 4:** The values of minimal inhibitory concentrations and minimal cidal concentrations (mg/mL) of cardamom ethanolic extract regarding tested microbial isolates.

	Ethanolic Extract (mg/mL)		Positive Control-Nystatin, (mg/mL)	
	MFC/MAC	MIC	MFC/MAC	MIC
*Candida albicans* HI	100	50	0.3	0.15
*Candida albicans* ATCC 24433	100	50	0.15	0.075
*Prototheca zopfii* HI	100	50	1.2	0.6
*Prototheca zopfii* AI	100	50	1.2	0.6

HI—isolate from human patient; ATCC—American Type Culture Collection; AI—isolate from animal; MFC—minimal fungicidal concentration, in mg/mL, refers to *Candida* strains; MIC—minimal inhibitory concentration, in mg/mL; MAC—minimal algaecidal concentration, in mg/mL, refers to *Prototheca* strains.

## Data Availability

The original contributions presented in this study are included in the article/Supplementary Materials. Further inquiries can be directed to the corresponding author.

## Supplementary Materials

The following Supporting Information can be downloaded at: https://www.mdpi.com/article/10.3390/molecules31142430/s1, Figure S1: Chromatogram of analyzed essential oil.

## Abbreviations

The following abbreviations are used in this manuscript:

AAE	ascorbic acid equivalents
AI	animal isolate
ATCC	American Type Culture Collection
DE	dry extract
DPPH	2,2-diphenyl-1-picrylhydrazyl
EO	essential oil
FRAP	ferric reducing antioxidant power
GAE	gallic acid equivalents
HbA1C	glycated hemoglobin
HI	human isolate
HOMA-IR	homeostatic model assessment for insulin resistance
IC_50_	inhibitory concentration which inhibits 50% of enzyme’s activity
MAC	minimal algaecidal concentration
MFC	minimal fungicidal concentration
MIC	minimal inhibitory concentration
MS	mass spectroscopy
QE	quercetin equivalents
RI	retention index
RSC_50_	radical scavenging concentration which inhibits 50% of generated radicals
RT	retention time
