# Supplementary figures and images for: Queen of Spices, Cardamom (Elettaria cardamomum (L.) Maton, Zingiberaceae)—In Vitro Assessment of Biological Potential
Source: Molecules. 2026 Jul 11;31(14):2430. doi: 10.3390/molecules31142430 (PMC13414071; doi:10.3390/molecules31142430)

Abundance

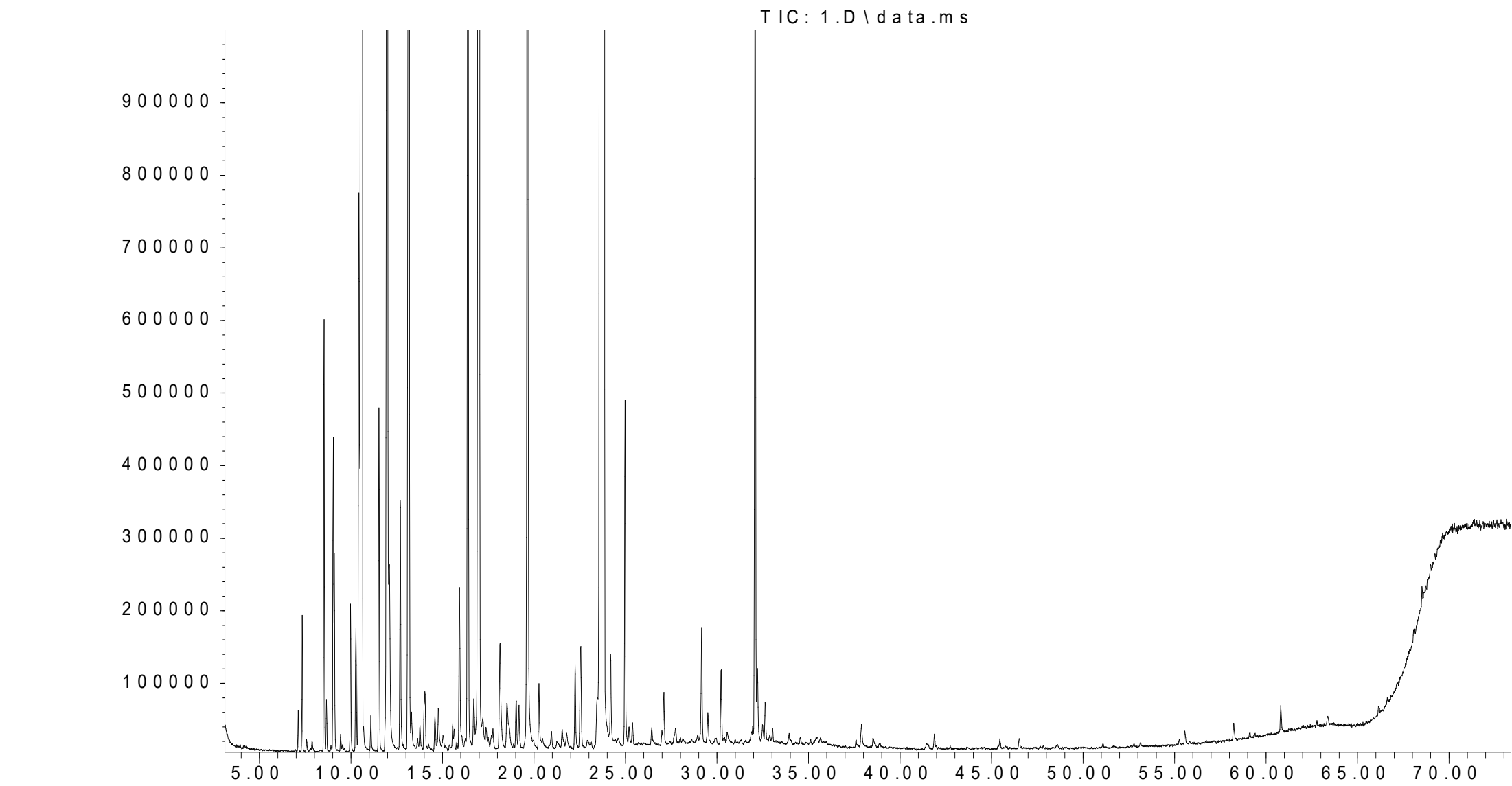

Time -->

Supplement: Supplementary file 1 [file molecules-31-02430-s001.zip › molecules-4377852-supplementary.pdf]
